# Curcumin and Its Modified Formulations on Inflammatory Bowel Disease (IBD): The Story So Far and Future Outlook

**DOI:** 10.3390/pharmaceutics13040484

**Published:** 2021-04-02

**Authors:** Adhimoolam Karthikeyan, Kim Na Young, Mohammad Moniruzzaman, Anteneh Marelign Beyene, Kyoungtag Do, Senthil Kalaiselvi, Taesun Min

**Affiliations:** 1 Subtropical Horticulture Research Institute, Jeju National University, Jeju 63243, Korea; karthik2373@gmail.com; 2 Department of Animal Biotechnology, Jeju International Animal Research Center (JIA) and Sustainable Agriculture Research Institute (SARI), Jeju National University, Jeju 63243, Korea; skdud559@naver.com (K.N.Y.); monir1983@jejunu.ac.kr (M.M.); antethesecond@gmail.com (A.M.B.); challengekt@jejunu.ac.kr (K.D.); 3 Department of Biochemistry, Biotechnology and Bioinformatics, Avinashilingam Institute for Home Science and Higher Education for Women, Coimbatore 641043, Tamil Nadu, India; kalaiselvi_bc@avinuty.ac.in

**Keywords:** crohn’s disease, ulcerative colitis, anti-inflammatory, curcumin nanoformulations, turmeric

## Abstract

Inflammatory bowel disease (IBD) is a chronic relapsing and remitting inflammatory disorder of the small intestine and colon. IBD includes ulcerative colitis (UC) and Crohn’s disease (CD), and it is a major factor for the development of colon cancer, referred to as colitis-associated cancer (CAC). The current treatment of IBD mainly includes the use of synthetic drugs and monoclonal antibodies. However, these drugs have side effects over long-term use, and the high relapse rate restricts their application. In the recent past, many studies had witnessed a surge in applying plant-derived products to manage various diseases, including IBD. Curcumin is a bioactive component derived from a rhizome of turmeric (*Curcuma longa*). Numerous in vitro and in vivo studies show that curcumin may interact with many cellular targets (NF-κB, JAKs/STATs, MAPKs, TNF-γ, IL-6, PPARγ, and TRPV1) and effectively reduce the progression of IBD with promising results. Thus, curcumin is a potential therapeutic agent for patients with IBD once it significantly decreases clinical relapse in patients with quiescent IBD. This review aims to summarize recent advances and provide a comprehensive picture of curcumin’s effectiveness in IBD and offer our view on future research on curcumin in IBD treatment.

## 1. Introduction

Inflammatory bowel disease (IBD) is a chronic relapsing and remitting inflammatory disorder of the small intestine and colon. Ulcerative colitis (UC) and Crohn’s disease (CD) are the two main types of IBD. UC is described through inflammation that is restricted to the colon. It starts in the rectum, incessantly extends to the nearby region, and often includes the periappendiceal site. However, the CD can involve any part of the gastrointestinal tract, generally, the terminal ileum or the perianal region, and 65% of the CD patients are affected in the small intestine’s lower end [1,2,3]. Both CD and UC are associated with enhanced risk of cancer, with period and severity of chronic colitis conferring major risk factors for colon cancer development, referred to as colitis-associated cancer (CAC) [4,5]. The characteristics of UC and CD are presented in Table 1. An imbalance of immune response, CD4^+^ Th1 to type 2 Th2 in favor of Th1 cells, appears to be an important pathogenic mechanism in IBD. This hypothesis is supported by research on IBD patients, where there is an increased number of proinflammatory cytokines, chemokines, and adhesion molecules in their mucosa [6,7].

The intestinal tight junction’s protein complexes link the atypical membranes between intestinal epithelial cells and act as a physical barrier, giving selective permeability. Deficiencies in tight junctions result in increased gut permeability, leading to an increased entrance of luminal antigens and contributing to intestinal inflammation [8]. IBD is one of the two most widespread diseases reflecting immune dysfunction, and another one is rheumatoid arthritis. The present epidemiological data show that the worldwide occurrence of IBD ranges between 12–20%, and incidence of IBD is increasing (150–250/100,000 population), particularly in developed countries. Family history is associated with a menacing issue for the progress of IBD, with a severe occurrence in the early stage of adult life. However, people of any age group can be affected [2]. The most common IBD symptoms include abdominal pain, blocked bowels, diarrhea, loss of body fluids, and weight loss. IBD is believed to be the result of the continuous inflammatory process mounted against endogenous microbes in a genetically predisposed individual. The etiology of IBD is not entirely understood. However, the existing studies revealed that IBD outcome is a result of a complex interplay between genetic, immunologic, and modifiable environmental factors in a genetically susceptible host against a subset of gut commensal microbiota [9,10]. The increasing rate of IBD points out the necessity of studying the environmental factors involved. Furthermore, knowing the environmental factors helps to prevent and treat the disease. Anti-inflammatory drugs (aminosalicylates, biological agents (i.e., anti-TNF monoclonal antibodies), corticosteroids, and immunosuppressants) are widely used to treat the IBD. However, the adverse effects (i.e., diarrhea and lymphopenia) extend time for treatment, and the high relapse rate limit their use. Systemic administration of anti-inflammatory drugs is the main reason for the adverse effects [11,12]. A potential method to treat the IBD is a precise target of the inflamed colonic region by routing the drugs orally to reduce side effects and increase the therapeutic potential.

Many studies witnessed a surge in using plant-derived products to manage various diseases, including IBD. Curcumin derived from *Curcuma longa* L. is involved in many important genetic and biochemical pathways and produces protective effects against several diseases. It is a harmless natural compound and also a potential therapeutic drug against various diseases [13,14,15,16]. Extensive research evidence supports the anti-inflammatory, antioxidant, antitumor, antimicrobial, and wound-healing effects of curcumin [17,18,19,20,21]. It is associated with many cellular targets (i.e., NF-κB, JAKs/STATs, MAPKs, TNF-γ, IL-6, PPARγ, and TRPV1) that effectively reduce the progression of IBD with promising results [22,23,24,25,26,27]. The study of curcumin and its modified formulations for IBD treatment has flourished over the decade. Many researchers have shown that curcumin has positive effects on IBD treatment [28,29,30,31,32]. In this review, we first compiled a brief introduction of IBD genetics and pathogenesis and curcumin molecular targets in IBD. Secondly, we wanted to explore the existing studies on curcumin and its modified formulations in IBD treatment, including clinical trials. Eventually, we wanted to discuss the major obstacles and provide our perspective on future research on curcumin in IBD treatment.

## 2. Genetics and Pathogenesis of Inflammatory Bowel Disease

**Figure 1:**
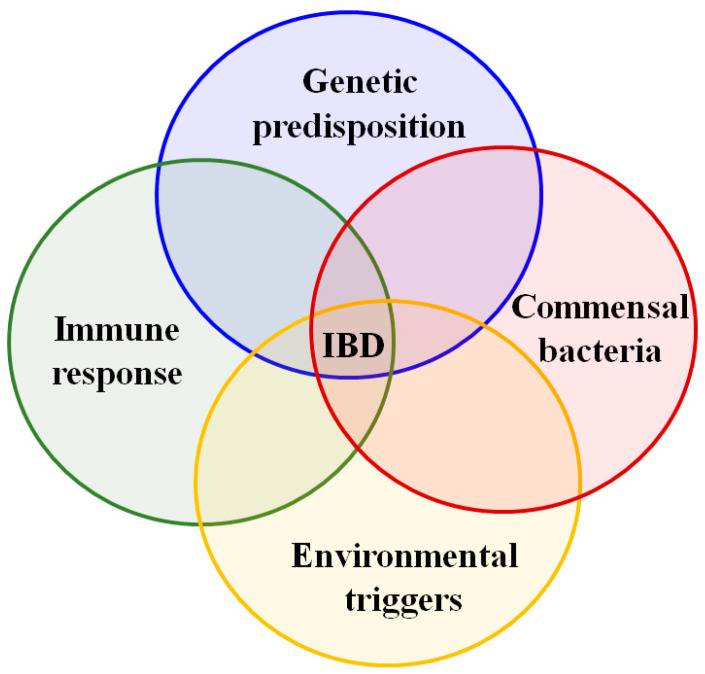
Etiology theories in inflammatory bowel disease (IBD), adapted from [9,10].

The major factors related to the IBD pathogenesis are presented in Figure 1 and Table 2. The genetics and pathogenesis of IBD are not well-understood yet. However, earlier studies have detailed that intestinal inflammation is increased or persists due to the non-adopted immune responses caused by interactions between genetic factors, gut microbiota, and environmental factors leading to the IBD [36]. In recent decades, we have seen that the research investigations of IBD genetics have increased and significantly improved our knowledge of IBD pathogenesis. It is mainly the recent technological advances in genome sequencing and genetic tests that permitted numerous association studies (genome-wide association studies, GWAS). Many studies have focused on patients in regions with high prevalence. Other studies are continuing worldwide with regions where IBD incidence is quickly growing. GWAS resulted in many susceptibility loci for IBDs in Caucasian populations (North America, Europe, and Israel). About 75,000 patients and controls were subjected to GWAS, and >163 susceptible loci have been identified [3]. Liu et al. [37] reported 38 new loci for IBD and showed the effectiveness of trans-ancestry association studies for mapping loci related to complex diseases. In another study, high coverage sequencing of 131 genes associated with CD in 500 patients from South Korea and the control group (1000 healthy individuals) identified eight new risk loci and three reported loci [38]. Considerable data given by previous population-based cohort studies showed that the disease risk increased 8–10-fold in people with immediate family history of UC or CD. Furthermore, studies concerning family history and twins have advocated that the possibility of emerging CD in another sibling while a child is suffering from CD increases 26-fold, compared to a 9-fold increase in UC [39]. Collectively, the study results show that genetic factors are involved significantly in the IBD predisposition.

About two hundred susceptible loci associated with IBD have been reported in the human genome [40]. Of these, 137 loci are associated with both UC and CD (referred to as IBD loci), whereas 37 loci are specific to CD and the remainder loci are specific to UC. Many common disease loci across divergent populations show clinical and pathological resemblance in UC and CD [41]. Hence, it is very likely that environmental and microbial factors play a more prominent role in shaping the disease’s expression in terms of CD versus UC. The growing number of susceptible loci for IBD has revealed that genetic mechanisms are the key factors associated with the disease pathogenesis. Moreover, the known genetic factors reason for the small changes in the diseases, CD (13.1%) and UC (8.2%). Collectively, to date, the identified susceptible loci and factors associated with genetic risk have shown 20–25% of heritability [37,42,43].

The shared loci between UC and CD suggest that the same genes and pathways are involved in the two diseases and a mechanistic continuum could be part of it. The studies involved in IBD loci unveil many pathways that are crucial for intestinal immune homeostasis, barrier function, epithelial restitution, microbial resistance, innate immunity regulation, reactive oxygen species (ROS) production, autophagy, adaptive immunity regulation, endoplasmic reticulum (ER) stress, and metabolic pathways associated with cellular homeostasis. IBD-associated genes are categorized into different groups and act individually on diverse barriers of the inflammatory pathways that include pathogen recognition, pathogen clearance by innate and cell-mediated immunities, and hindrance of pathogen invasion by the intestinal mucosal barrier. Among the reported genes, few genes, including NOD2, ATG16L1, IRGM, LRRK2, PTPN2, IL23R, Il10, Il10RA, Il10RB CDH1, and HNF4α, are well-studied [44]. NOD2 was the first CD-associated gene identified more than a decade ago. It involves as an intracellular receptor for monocytes bacterial products and causes signals for NF-kB activation. The NOD2 activation together with muramyl dipeptide causes autophagy in dendritic cells. The susceptible variants in NOD2 lacking in autophagy induction are found in the CD patients’ dendritic cells. However, decreased bacteria localization is exhibited in autophagolysosomes [45]. Two autophagy-associated genes (ATG16L1 and IRGM) in the host innate immune response in CD slowly fade away and are now covered by ATG4, a novel discovered gene that promotes the autophagy process in CD patients. The genetics identified to confer an increased risk of CD explain the need for innate immunity, autophagy, and phagocytosis in its pathogenesis. IL23R and PTPN2 genes associated with the autoimmune disorder demonstrate another aspect of CD pathogenesis. The genes that can control T cell responses are AHR, CCL20, CD28, LY75, NFATC1, and NFKBIZ [37].

Integration of gene mapping and exome sequencing disclosed a set of rare genetic variants related to early-onset IBD (VEO-IBD) [46]. For instance, CTLA-4, a new variant discovered by Zeissig et al. [47], is related to autoimmunity and early-onset CD. Another study identified that VEO-IBD patients carry rare heterozygous missense variants in IL10RA and novel variants in genes (i.e., MSH5 and CD19) associated with immunodeficiency [46]. The rare NCF2 variant has been reported in 4% of VEO-IBD patients compared to 0.2% of controls. It decreased the binding of proteins and part of oxidase activity inhibition [48]. IL-10 mainly regulates anti-inflammatory activities and sustains intestinal mucosal homeostasis in the gastrointestinal tract. When IL-10 binds with the tetrameric receptor complex, it stimulates Tyk2 and JAK1, leading to the phosphorylation of signal transducer STAT-3 and transcription of downstream target genes, eventually upholding the anti-inflammatory effector’s expression [49]. Moreover, IL-10 or IL-10 receptor mutations result in a severe IBD at an early age [50].

The existing association studies have shown that there is a considerable amount of heritability which cannot be explained. This missing inheritance also results from additional common variants (likely many more with very small impact) or rare variants. It has recently been found that mostly rare alleles (i.e., NOD2 and CARD9) contribute to common disease genetics [42]. There are not many variables that can predict the initiation and progression of IBD. The findings of IBD genetic research may help clinical practices identify the subphenotype of IBD and predict the disease development and prognosis, allowing for the detection of patients who may benefit from early aggressive treatment due to their poor prognosis. Jostins et al. [51] examined the genetic architecture of IBD and detailed the overlapping between IBD-susceptible loci and other diseases. A substantial amount of the loci identified is the same between IBD and other complex diseases (immune-arbitrated diseases, primary immunodeficiency, Mendelian susceptibility to mycobacterial disease, and leprosy). IBD and mycobacterial infections share common susceptible loci such as IL12B, IFNGR2, STAT1, TYK2, STAT3, IRF8, and IFNGR1. Furthermore, NOD2, C13orf31, LRRK2, RIPK2, TNFsf15, and IL23R are other susceptible loci that characterize both IBD and leprosy. Anti-TNF is a potential candidate for IBD treatment, also linked to latent mycobacterial disease recrudescence. This information could be helpful to understand treatment-associated adverse effects [52].

## 3. Curcumin’s Molecular Targets in Inflammatory Bowel Disease

**Figure 2:**
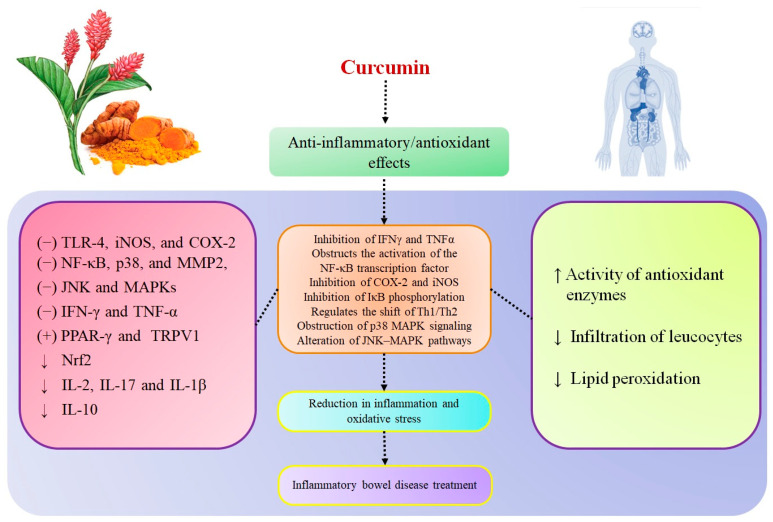
Curcumin’s potential and molecular targets in inflammatory bowel disease (IBD) treatment, adapted from [25,66,67,68,69,70,71,72,73,74].

Curcumin is a promising candidate that has the ability to manage or control IBD. Many studies have shown curcumin efficacy in IBD treatment based on its anti-inflammatory and antioxidant effects. Here, we outlined the curcumin potential and molecular targets in IBD treatment (Figure 2). Neutrophils are primary proinflammatory effector cells able to connect with lymphocytes to foster epithelial dysfunction and injury related to IBD. Curcumin efficiently decreases the neutrophils infiltration to the inflammatory sites by disturbing the development of a chemokine gradient as well as by direct effects of the compound on neutrophil polarization, chemotaxis, and chemokinesis. These mechanisms are mainly involved in curcumin’s protective effect at the time of intestinal inflammation [60,61]. Curcumin controls inflammation by downregulating the genes related to oxidative stress and fibrogenesis pathways. The activity of PI3K and phosphorylation of AKT assist in decreasing cell death. At the same time, curcumin obstructs neutrophils and downregulates PI3K and AKT phosphorylation [60]. Curcumin efficiently decreases the activity of proinflammatory cytokines (i.e., IFN-γ, TNF-α, IL-1, and IL-8) through communication with many transcriptions and signaling molecules (i.e., NF-κB, JAKs/STASs, MAPKs, and β-catenin) [62,63,64,65].

**Figure 3:**
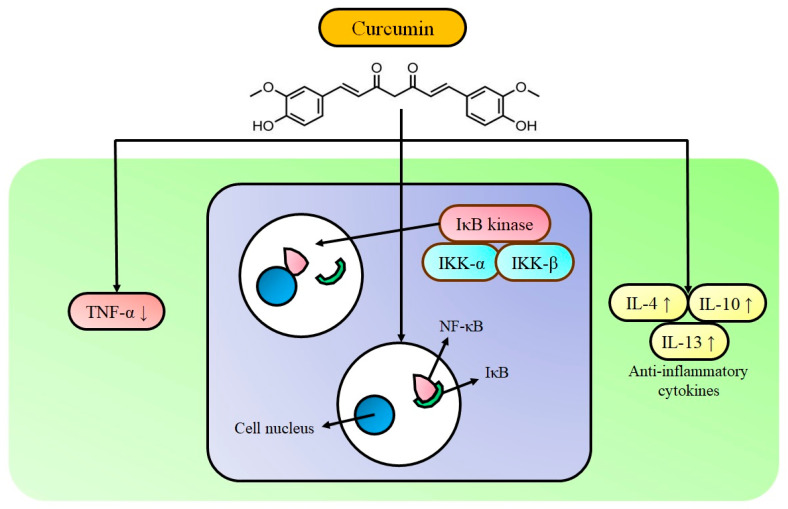
Curcumin’s activity against IBD: inhibition of the activation of NF-κB, adapted from [75].

Many studies have shown that NF-κB inhibitory activity is studied as the primary target for IBD intervention [75]. Curcumin’s action against IBD for inhibiting the activation of NF-κB is presented in Figure 3. Curcumin obstructs the NF-κB expression by modulating the NF-κB/IκB pathway. IκB phosphorylation at serine 32 and 36 is required for its degradation and further NF-κB activation. Curcumin prevents IκB degradation therefore obstructing NF-κB activation and disturbs upstream signaling of the NF-κB-inducing kinase and the IκB kinase [76]. Experimental results from Atreya et al. [77] showed that the NF-κB p65 level was higher in colon biopsies of patients. The severity of intestinal inflammation and the quantity of NF-κB p65 was associated with tissue samples. A high level of NF-κB expression increased the capability to secrete cytokines (i.e., TNF-α, IL-1, IL-6, IL-12, and IL-23). It was associated with mucosal damage in IBD. Curcumin decreases the expression level of TNF-α, an important cytokine associated with the inflammatory cascade of IBD, and it effectively reduces the oxidative stress initiated by TNF-α [77]. Studies have also demonstrated that curcumin plays the role of an IFN-γ signaling inhibitor in colonic epithelial cells with a mechanism of biphasic action. It provides beneficial effects of curcumin to manage the IBD [78]. Curcumin is able to downregulate the expression of Th1 and nitric oxide production. Thereby, it can control the activation of macrophages. It improves the peritoneal macrophage phagocytosis and differentially regulates the splenocyte proliferation [79,80]. Curcumin has been shown to suppress inflammation by selectively hindering COX-2 receptors [81]. Its anti-inflammatory activities are often correlated with a decrease in the Th1 activity, contributing to iNOS and lipid peroxidation suppression and eventually decreasing tissue injury. Furthermore, the increasing number of ROS scavengers in the colon suggests that ROS also has significant involvement in IBD-like reactive nitrogen species (RNS) pathophysiology. Several studies showed that curcumin’s treatment efficiently decreases the malondialdehyde, nitrogen oxide, serine protease, and superoxide anions in the colonic mucosa, confirming the curcumin’s beneficial effects on IBD [72].

## 4. Curcumin in Inflammatory Bowel Disease

NF-κB is a vital transcription factor associated with cytokine and chemokine production and is essential for inflammation. Therefore, many treatments used for IBD target NF-κB. One study indicated that curcumin obstructed NF-κB activation and decreased the macroscopic damage scores in a dinitrobenzene sulfonic acid (DNBS)-induced colitis model [82]. The curcumin’s effect was further confirmed by a decrease in myeloperoxidase activity, gene expression analysis, and a decline of the DNBS-induced message for IL-1β. Furthermore, curcumin decreased the reproducible dinitrobenzene (DNB)-induced p38 MAPK signal activation in intestinal lysates [82]. Venkataranganna [83] posited that the curcumin’s inhibitory effect improves intestinal oxidative stress and reduces the expression of NF-κB and iNOS, thus an improvement of colonic damage in 2,4-dinitrochlorobenzene (DNCB)-induced colitis rats was observed. This study revealed that an inhibitory effect of curcumin depends on the dosage. It protected DNCB-prompted changes in the colon length and body weight based on curcumin dose and increased the MPO, LPO, and ALP levels induced by DNCB.

MAPKs, including p38 MAPKs and JNK, play a significant role in regulating the transcription of several genes associated with inflammation. To study the curcumin’s mechanism in chronic colitis rats, 50–100 mg/kg of curcumin was routed orally to a trinitrobenzene sulfonic acid (TNBS)-induced rat model daily for two weeks [84]. The experimental results revealed that curcumin effectively attenuated damage and activity of TNF-α and myeloperoxidase. Furthermore, it decreased the nitrite level and iNOS/COX-2 expression and attenuated the p38 MAPK activation. However, at the same time, no significant differences were obtained in JNK activation. Collectively, the results showed that curcumin effectively reduced the growth of chronic experimental colitis. TRPV1 is exhibiting a protective role after the initiation of intestinal inflammation made through DNBS in mice. Furthermore, immunoreactivity of TRPV1 was highly increased in colonic nerve fibers of IBD patients. A study by Martelli et al. demonstrated that curcumin could interact with the TRPV1 receptor to regulate its protective role in DNBS-induced colitis. However, the same research also defined that this protective role was ended by pretreatment with capsazepine, a TRPV1 antagonist [85].

Curcumin effect in TNBS-induced colitis NKT-deficient SJL/J mice (Th1-mediated inflammation) and BALB/c mice (Th1/Th2 mixed response) studied by Larmonier et al. Curcumin efficiently increased survival, controlled body weight loss and disease index. Interestingly, no protective effects were obtained in the SJL/J mice. Furthermore, a microarray study of colonic gene expression defined that the two strains exhibited different reactions to curcumin. The study outcomes show that the dietary curcumin’s therapeutic potential could be varied based on the nature of immune dysregulation in IBD [67]. As discussed earlier, inhibition of NF-κB has been reported as a potential target for intervention in IBD. NF-κB is induced by many pathways. Of these, tracing upstream signaling pathways is considered an effective approach for the treatment of IBD. Lubbad et al. used curcumin in an experimental colitis-induced rat model through intrarectal administration of TNBS. They studied the upstream signaling pathways via analyzing the expression of TLR-4 and MyD88. Curcumin treatment suppressed the expression level of proteins viz., TLR-4, MyD88, and NF-κB in the inflamed tissue and suggested that upstream signaling molecules TLR-4 and MyD88 are the potential therapeutic target in IBD [86]. In another study to investigate the significant disease mediators in the gut of children and adults with IBD, the colonic mucosal biopsies (CMB) and colonic myofibroblasts (CMF) were cultured ex vivo with curcumin. Curcumin treatment attenuated p38 MAPK activation, improved IL-10, and decreased IL-1b activity. Furthermore, curcumin efficiently suppressed MMP-3 in CMF in a dose-dependent manner [87].

Motawi et al. stated that curcumin considerably decreased the seriousness and degree of damage induced by TNBS colitis. Curcumin decreased MMPs (MMP-1, MMP-3, and TIMP-1) expression and nitric oxide production and downregulated the iNOS protein expression [88]. Miller et al. first reported the curcumin’s ability to enhance the malignant mesothelioma cells killing by induction of pyroptosis without activation of cytokines due to the obstruction of TLR and NF-κB pathways. This study provides evidence that curcumin warrants further investigation as a therapeutic agent in malignant mesothelioma cells [89]. The turmeric extract was also investigated for the value of genetic predisposition to unsuitable inflammation. McCann et al. studied the turmeric extract’s effect on the functionality of the SLC22A4 and IL-10 variants associated with IBD in HEK293 cells. The results showed that the turmeric extract significantly attenuated SLC22A4 and IL-10 by reducing the inappropriate epithelial cell transport and improving the cytokine gene promoter level related to the anti-inflammatory activity [90].

Curcumin has exhibited promising therapeutic effects in a dextran sulfate sodium (DSS)-induced colitis mouse model by obstructing the p38MAPK activity, decreasing the proinflammatory cytokines (i.e., TNF-α) production, and neutrophil infiltration. Furthermore, curcumin decreases the injury caused by inflammation in the mouse intestines and exhibits its capability in UC therapy [73]. In another study, curcumin reduced colon growth with partial effects on mucosal immune responses. AOM/Il102/2 mice (colitis-associated colorectal cancer model) were administered curcumin for six weeks. Curcumin commendably increased the survival rate and reduced the colon weight/length ratio. In contrast, no changes were observed in the PBS-treated control. At the same time, curcumin controlled the age-related reduction in alpha diversity and increased the colonic histology, bacterial richness, and the amount of *lactobacilli* in AOM/Il102/2 [31]. Many studies have detailed the role of dendritic cells in the initiation of pathogenesis in IBD. They are mediated by several signaling pathways such as JAK, STAT, and SOCS. Zhao et al. demonstrated the curcumin’s anti-inflammatory mechanism in a TNBS-induced colitis mice model by studying the activation of dendritic cells JAK, STAT, and SOCS signaling pathways. The significant outcome of this study was the inhibition of phosphorylation of the three members (JAK2, STAT3, and STAT6) of the JAK, STAT, and SOCS signaling pathways and the higher expression of three downstream proteins including SOCS1, SOCS3, and PIAS3 on these pathways. This study also explained how curcumin effectively decreases dendritic cell activation to regain immunologic balance to treat colitis efficiently [78].

Cooney et al. detailed the curcumin’s effect in colon inflammation in a Mdr1a−/− mouse model of human IBD through the combination of transcriptomic and proteomic data. The results showed that the Mdr1a−/− mouse colon might be mediated by α-catenin activation, which had not previously been reported. The study presented the evidence to support the curcumin’s effect by multiple molecular pathways such as reduced immune response, increased xenobiotic metabolism, resolution of inflammation by decreased neutrophil migration, and improved barrier remodeling. Besides, the key transcription factors and other regulatory molecules (ERK, FN1, TNFSF12, and the PI3K complex) activated in inflammation were downregulated by dietary intervention with curcumin [45]. Yang et al. showed the curcumin’s therapeutic benefits for inflammation in a rat model with UC. Curcumin reduced the disease activity index (DAI) and histological changes in the colon and downregulated the expression of transient receptor potential vanilloid 1 (TRPV1) in DSS-induced colitis rats [91]. IFN-γ is a major proinflammatory cytokine related to IBD pathogenesis. IFN-γ-treated HT29 cells resulted in apoptotic changes that could reproduce the damage of intestinal epithelia exposed to inflammatory cytokines. HT29 cells were pretreated with curcumin, then stimulated with IFN-γ. The results showed that curcumin effectively reduced the secretion of IL-7 and suggested the role of curcumin in IBD [92]. Curcumin or tetrahydrocurcumin was routed to an experimental colitis animal model. The results showed that curcumin effectively decreased severity of the DSS-induced colitis in an animal model by reducing NF-κB/STAT3 activation and iNOS/COX-2 proteins expression. However, tetrahydrocurcumin produced weaker inhibitory effects than curcumin [93].

The NLRP3 inflammasome is an important one for IBD development due to its mediating role in IL-1β maturation. The experimental results obtained by Gong et al. revealed that curcumin intervention obstructed the activation of the NLRP3 inflammasome in a DSS-induced colitis model. Curcumin controlled weight loss, reduced the disease score, and maintained the colon length. It attenuated the inflammatory cytokines (i.e., IL-1β and IL-6), MCP-1 expression levels, MPO and caspase-1 activities, and histopathological damage. The outcome from this study supports the statement that curcumin suppresses the activation of NLRP3 and ameliorates DSS-induced colitis in mice. Therefore, curcumin is a suitable drug for clinical application for IBD treatment [94].

Similarly, in another study, curcumin treatment decreased the DAI and histopathological score in a DSS-induced colitis mice model by inhibiting excessive autophagy and regulation of cytokine networks [95]. Zhang et al. developed a UC model by giving mice 3.5% DSS for seven days and then studied the curcumin effect in DSS-induced mice. The impact of DSS was confirmed by macroscopic and microscopic alterations that occurred in mice. Curcumin (50 mg/kg) treatment showed a better survival rate of the mice with UC. It controlled the loss of body weight and disease severity better than in the DSS-treated mice. Curcumin effectively disturbed the colonic architecture and decreased proinflammatory cytokine activity. Furthermore, it reduced the expression of autophagy‑associated 12, Beclin‑1, and microtubule-related proteins (light chain 3 II). It increased the phosphorylated mTOR and SIRT1 expression level in the colon tissue compared to the DSS-induced group. Collectively, this study revealed that curcumin’s clinical activity in DSS‑induced UC was partly suppressed by the intestinal inflammatory cascade response, autophagy reduction, and SIRT1/mTOR signal control [96]. Wei et al. conducted a study on the curcumin’s therapeutic mechanism in UC. Curcumin was orally routed to DSS-induced colitis mice for seven days. The experimental results showed that curcumin effectively reduced the disease index and the spleen index and improved the mucosal inflammation. Curcumin controlled the re-equilibration of Treg/Th17 and attenuated the IL‑6, IL‑17, and IL‑23 expression levels, but increased the IL-10 expression level in the colon. Besides, no considerable changes were observed in HIF1A between the colitis group and the curcumin group. Collectively, the results suggest that curcumin can treat colitis by regulating the re-equilibration of Treg/Th17 and that the regulatory mechanism could be associated with the IL‑23/Th17 pathway [97]. The details of in vitro and animal studies of curcumin administration in IBD treatment are presented in Table 3.

## 5. Curcumin Combinations with Other Therapeutic Molecules and Modified Curcumin Formulations in Inflammatory Bowel Disease

Many studies have suggested that the liposome encapsulation of curcumin permits systemic administration. The research outcomes obtained by Li et al. confirmed that treatment with liposomal curcumin induced a dose-based growth obstruction in human colon cell lines LoVo and Colo205. The in vivo studies demonstrated the effects of liposomal curcumin by attenuating the expression level of CD31 (an endothelial marker), IL-8, and the vascular endothelial growth factor in an animal model [99]. Yadav et al. evaluated curcumin cyclodextrin complex’s anti-inflammatory effects for treating IBD in a colitis-induced rat model. Treatment with this complex resulted in a weight gain in rats, notably, the colon length was longer compared to rats in the standard curcumin and DSS control groups. Collectively, the study results suggested that the colitis induced in rats by supplementation of DSS was significantly reduced by curcumin cyclodextrin complex [100]. The same research group prepared solid lipid microparticles of curcumin (SLM_C) and studied the anti-inflammatory effect to treat IBD in a colitis-induced rat model by a colon-targeted release method. In this study, the authors developed SLM_C with many lipids (i.e., palmitic acid, stearic acid, and soy lecithin using an adjusted percentage of poloxamer 188). The results were similar to the previous reports. The increase in weight and colon length was observed in solid lipid microparticles of curcumin compared to native curcumin and DSS control rats. SLM_C considerably decreased the degree of DSS-induced colitis [101].

Finding and using effective therapeutic molecules or their combinations is another first-line treatment for UC. Celecoxib (anti-inflammatory drug) is gaining attention and is a better choice to combine with curcumin to alleviate the UC. The pH-sensitive Eudragit R S100 nanoparticles of curcumin–celecoxib were formulated and studied for their effectiveness in a TNBS-induced UC rat model. It revealed that the combination of curcumin nanoparticles with celecoxib was much more effective compared to nanoparticles of either product or the drug suspension. At the same time, nanoparticles alone could not show beneficial effects. Thus, verifying the benefits of combining drugs (curcumin + celecoxib) helps UC treatment [102].

Sareen et al. performed an in vivo study to test the native curcumin and curcumin-loaded microsponges’ effects in an acetic acid-induced colitis rat model. The study results showed that an optimized formulation resulted in reduced edema, necrosis, and colon hemorrhage compared to native curcumin [103]. They suggested that curcumin microsponges can be used in the drug delivery system in the treatment of UC. Beloqui et al. developed curcumin nanoparticles by combining poly (lactic-*co*-glycolic acid) (PLGA) and a polymethacrylate polymer (Eudragit1 S100). In this study, curcumin nanoparticles resulted in an increased curcumin penetration in monolayers compared to curcumin in a suspension. It decreased the TNF-α secretion on LPS-stimulated macrophages. The in vivo result showed that curcumin nanoparticles reduce the neutrophil infiltration and TNF-α secretion. However, it keeps the colon structure the same as in the control group in a murine DSS-induced colitis model. These findings revealed that the combination of PLGA and Eudragit1 S100 is better for curcumin delivery in the treatment of IBD [29]. Likewise, fabricated microparticles (MPs) with pH-sensitive Eudragit S100 (ERS100) and poly (lactic-co-glycolic acid) (PLGA) were used to formulate the curcumin-loaded microparticles to increase the loading efficiency, sustain release of curcumin, and target colon cells. The authors also reported that orally routed curcumin microparticles had a stronger therapeutic potential and anti-colitis activity in a UC mouse model than unformulated curcumin [104].

Curcumin and piperine were used to co-encapsulate in the nanoformulation called self-microemulsifying drug delivery system (SMEDDS) and prepare a CUR-PIP-SMEDDS to increase curcumin stability/water solubility and alleviate colitis in a UC model. The mean size of a CUR-PIP-SMEDDS was 15.87 ± 0.76 nm. The drug encapsulation efficacy of SMEDDS for curcumin and piperine was 94.34 ± 2.18 and 90.78 ± 2.56, respectively. Furthermore, CUR-PIP-SMEDDS had a greater therapeutic efficacy in ameliorating colitis in a DSS-induced model compared to curcumin. These findings showed that a CUR-PIP-SMEDDS was a possible carrier for generating a colon-targeted drug delivery system of curcumin for the management of UC [105].

In recent years, the focus on target drug release to swollen tissues attracted extensive attention for IBD treatment. Combining anti-inflammatory drugs and lipids at the same time inside a nanocarrier might be a potential option for the management of IBD. Beloqui et al. synthesized three lipid-derived nanocarriers (self-nanoemulsifying drug delivery systems (SNEDDS), nanostructured lipid carriers (NLC), and lipid core–shell protamine nanocapsules (NC)) having curcumin and compared their potential for IBD treatment. Though NCs were comparable to SNEDDS (NC > SNEDDS > NLC and curcumin (CC) suspension), a 30-fold greater curcumin absorbency across Caco-2 cell monolayers was attained in an in vitro study. CC SNEDDS and CC NLCs potentially decreased the activity of TNF-α in LPS-stimulated macrophages. However, no such effect was seen for CC NCs or the curcumin suspension. On the other hand, in vivo, CC NLCs alone decreased the TNF-α secretion and neutrophil infiltration and reduced the inflammation in a DSS-induced murine colitis model [30]. Mutalik et al. detailed the development of a drug (grafted copolymeric nanoparticles loaded with curcumin) appropriate for targeting the colon [106]. The researchers synthesized a new formulation of pH-sensitive hydrolyzed polyacrylamide-grafted xanthan gum (PAAm-g-XG) nanoparticles (NP) loaded with curcumin. The study outcomes showed that the maximum amount of curcumin delivery was recorded after rat cecal contents were incorporated in a pH 6.8 solution. The formulated curcumin controlled the loss of body weight and reduced colonic inflammation by effectively decreasing myeloperoxidase and nitrite levels in acetic acid-induced IBD in rat models.

Eudragit S-100-coated chitosan microspheres of curcumin were developed through the emulsion cross-link method. It controlled the early release of curcumin and exhibited sustained release for up to 12 h in the Higuchi model. In contrast, uncoated curcumin chitosan microspheres showed burst release after 4 h. Furthermore, an in vivo study in an acetic acid-induced mice colitis model confirmed a significant decrease in severity and amount of damage in the colon with curcumin-loaded microspheres compared to free curcumin. It was further proved by histopathological analysis [107]. The microparticulate system is planned for intestine-targeted delivery of curcumin (CUR). The system developed using microspheres (Ms) based on zein (Z.N.) and Gantrez1 AN119 (PVMMA) together with a coating of a pH-sensitive polymer (Eudragit1 FS30D). Curcumin-loaded microspheres produced a major obstruction in cytokine (i.e., TNF-α, IL-1b, NOS2, and COX-2) activity in LPS-stimulated macrophages [108]. Huang et al. investigated the effect of curcumin with soybean oligosaccharides (CSO) on UC rat models by analyzing the intestinal flora. CSO treatment efficiently reduced the expression of TNF-α and IL-8. It also effectively decreased inflammation in the colonic mucosa and tissue damage compared to the control [109].

On the other hand, a number of curcumin analogs with various substitution groups (R=H–, Br–, Cl–, F–, NO_2_–, CH_3_–, and OH–) has been studied to substitute the methoxy group in in vitro and in vivo studies. Curcumin, CUR-OH, and CUR-Br (25 μM) reduced the nitrogen oxide production and expression of iNOS and COX-2 in the NF-κB signaling pathway in LPS-induced macrophage cells. In contrast, other analogs, in particular, CUR–NO_2_, were not effective. Based on the in vivo study in a DSS-induced colitis C57BL/6 mice model, the CUR-Br analog exhibited a positive effect similarly to curcumin, while the CUR-NO_2_ analog could not show any impact in the mice. Collectively, Yang et al. showed that the methoxy group is a possible structural candidate for developing curcumin-derived drugs for IBD [65]. Curcumin with essential turmeric oils referred to as ETO-curcumin and normal curcumin was examined in a DSS-induced colitis mice model. Interestingly, ETO-curcumin offers better anti-inflammatory activity than standard curcumin. It was proved by analyzing the gene expression of IL-10, IL-11, and FOXP3. All the genes expressed highly in the colon in response to ETO-curcumin treatment [110].

Nanoparticle curcumin (NC) decreases the progress in a DSS-induced colitis mice model by modulating gut microbial structure. It controls the body weight loss, reduces the disease’s severity, the histological colitis score, and enhances mucosal permeability. NC treatment inhibited activation of NF-κB in colonic epithelial cells via suppressing NF-κB [111]. Researchers formulated and characterized the amphiphilic curcumin polymer (PCur) (hydrophilic polyethylene glycol (PEG) and curcumin (CUR) connected through a disulfide bond). The resulting PCur particles have better solubility, nanosize, and near-neutral surface capability. These are the reasons for better accumulation of curcumin at swollen areas of the gut. Besides, PCur exhibited controlled drug release and improved robustness in the gastrointestinal tract’s physiological pH. Furthermore, a considerably higher drug release was seen once reacting to bacterial reduction in the colon. Eventually, oral administration of PCur reduced the colon’s inflammation development and effectively protected the mice from IBD [112].

Kesharwan et al. assessed effectiveness of the polymer–drug complex of curcumin (Ora-Curcumin-S) in preventing IBD. The coprecipitation method was used to develop Ora-Curcumin-S through hydrophilic polymer Eudragit^®^ S100. The formulation was then subjected to study the pharmacokinetic properties. It revealed that Ora-Curcumin-S increased the curcumin solubility and stability. At the same time, Ora-Curcumin-S exhibited the potential to precisely target curcumin in the colon tissue at the luminal side compared to native curcumin. Ora-Curcumin-S effectively obstructed the monophosphoryl lipid A/*E. coli*-induced inflammatory activity in dendritic cells by upregulating the TLR-4 expression, confirming that Ora-Curcumin-S is a polymer-based TLR-4 antagonist. The effect of Ora-Curcumin-S was further confirmed by an in vivo study on a mouse model of UC, which revealed the most significant prevention of colitis and associated injury [113]. This study provides a model of a polymer-curcumin-based TLR4 antagonist and preclinical data supporting local delivery and effectiveness of the long-sought clinical application of curcumin for controlling IBD pathobiology and CAC. Chen et al. [114] used the emulsion solvent evaporation method to construct bowl-shaped microparticles (BMPs) and load them with curcumin. Further, oral administration of BMPs loaded with curcumin exhibited excellent therapeutic effects and improved the DSS-induced UC mouse model.

Solid binary lipid nanoparticles (SBLN) were used to encapsulate curcumin to improve stability, loading efficiency, cellular uptake, and also therapeutic properties of curcumin. Curcumin-loaded SBLN were synthesized using the solvent emulsification evaporation method with the support of binary lipids. The resultant CUR-SBLNs pointedly enhanced the uptake and localization of cells during IBD in inflamed tissues. It was shown that oral administration of CUR-SBLNs reduced the infiltration, oxidative stress, and secretion of TNF-α in a DSS-induced colitis model. CUR-SBLNs also maintain the colon’s structure like in the healthy animal group (guinea pigs) compared with unformulated curcumin [32]. Szebeni et al. formulated Mannich curcuminoids (C142 or C150) and tested the anti-inflammatory activity in a lipopolysaccharide (LPS)-treated B16 melanoma cell line and a TNBS-induced colitis rat model. LPS cotreatment with C142 or C150 obstructed NF-κB activity in a dose-dependent manner. C142 or C150 exhibited an obstruction in major inflammatory cytokines such as TNF-α, IL-6, and IL-4 expression on LPS-stimulated cells. Treatment with C142 or C150 in a colitis rat model decreased infiltration of leukocytes in the submucosa and muscular propria of the inflamed gut. Furthermore, it controlled weight loss, reduced the severity of colonic inflammation and hemorrhagic lesion sizes [115].

In another research, porous poly (lactic-*co*-glycolic acid) nanoparticles were loaded with curcumin with the support of pluronic F127 (PF127). These porous PF127-functionalized curcumin-loaded nanoparticles were reported to show better biocompatibility and cellular accumulation of curcumin than porous curcumin-loaded nanoparticles without PF127 alteration, suggesting the involvement of porous PF127 in improving cellular uptake. In this study, researchers observed that porous PF127 nanoparticles obstruct IL-6, IL-12, and TNF-α expression more than nanoparticles and non-porous PF127 nanoparticles. An in vivo study was conducted on a UC mice model where the formulations were routed orally with porous PF127 nanoparticles showing better results than porous nanoparticles and non-porous PF127 nanoparticles. This study revealed porous PF127 nanoparticles have a great potential as an effective curcumin nanocarrier for UC treatment [116].

Curcumin chitosan microsphere (CCM) effects were studied in rats with UC induced by TNBS. Oral administration of CCM resulted in a low level of NF-κB expression and decreased the damage score in colonic mucosa. The serum levels of IL-1β and IL-6 in CCM-treated rats were considerably reduced (*p <* 0.01) as compared with the mesalazine enteric-coated tablets (MECT) group. In contrast, the IL-4 content was significantly higher than in the model control group (MCG) and MECT groups (*p <* 0.01). The results showed that CCM offers a promising therapeutic approach for treating UC in a clinical study [117].

Bioadhesive systems attract a mounting attention because of their potential to confine drug delivery together with sustained release. Recently, using this system, Desai and Monim demonstrated that the curcumin and cyclosporine combination exhibit a tremendous synergistic effect and potential for controlling IBD. It has a good impact when given in a minimum dose as compared to separate drugs given in high doses. Carbopol 940 (CP940) and hydroxypropyl cellulose (HPC-H) were used to develop the curcumin and cyclosporine bioadhesive pellet cores released to the colon in a controlled manner using the pH-sensitive polymer Eudragit^®^ S100. The drug-loaded pellets were evaluated in an acetic acid-induced UC rat model. Results showed that curcumin and cyclosporine pellet cores could improve the acetic acid-induced UC rat model. It was confirmed by analyzing the body weight, clinical parameters, macroscopic and microscopic parameters [118]. Oshi et al. formulated a colon-targeted core−shell using curcumin nanocrystals as cores and pH-responsive polyelectrolyte multilayers of chitosan/sodium alginate/cellulose acetate phthalate as shells (CAP1AG4CH5@CUNCs) to deliver a drug precisely into the inflamed colon in UC. CAP1AG4CH5@CUNCs feature the pH-dependent release of curcumin and high colonic distribution in mice in in vitro and biodistribution studies. Eventually, CAP1AG4CH5@CUNCs exhibited a better therapeutic potential in treating DSS-induced colitis in mice and were acknowledged as an excellent system to deliver drugs in the colon for UC treatment [119]. Luo et al. [120] synthesized a nanocarrier having a tannic acid (TA)-coated genipin (GNP)-crosslinked human serum albumin (HSA) to encapsulate curcumin and prepared curcumin nanoparticles (TA/CUR-NPs). The resulting nanoparticles extended curcumin’s colon adhesion and improved its uptake in Caco-2 cells.

Furthermore, oral administration of TA/CUR-NPs confirmed its anti-UC benefits by effectively decreasing colitis symptoms in a DSS-induced mice model through obstructing the TLR4-associated NF-κB signaling. P-CUR/CAT-NPs were obtained using poly (lactic-*co*-glycolic acid) (PLGA)-based nanoparticles (NPs) with pluronic F127 (PF127) and catalase (CAT)/curcumin (CUR). They improved the cellular uptake efficiency of macrophage cells. Moreover, P-CUR/CAT-NPs exhibited the ability to reduce the proinflammatory cytokine secretion as compared to other nanoparticles (CUR-NPs and P-CUR-NPs), suggesting that P-CAT/CUR-NPs are a better option for UC treatment [121]. In another investigation, Rotrekl et al. for the first time showed that glucan particles are a potential carrier for curcumin in IBD treatment. Curcumin with glucan particles efficiently reduced the TNF-α, IL-1β, and IL-6 activity as compared to the physical mixture of native curcumin and glucan particles in DSS-induced colitis in rats. It can also be able to decrease the activity of MMP-9 [122]. See the details of in vitro and animal studies of curcumin combinations with other therapeutic molecules and administration of modified curcumin formulations for IBD treatment in Table 4.

## 6. Clinical Trials

So far, many clinical trials have been conducted to identify safety and effectiveness of curcumin in the management of IBD. People of different age groups participated in these clinical trials. The number of male and female patients who participated in clinical trials did not vary significantly. The clinical trials were conducted in single or multiple centers using a placebo-controlled randomized double-blind study design. They used different doses and formulations of curcumin and different administration routes. Various methods were followed to examine the clinical activity of the disease and the endoscopic scores. Many studies showed positive outcomes after the curcumin intervention in IBD. Several clinical trials conducted with UC and CD patients are discussed and outlined here.

### 6.1. Ulcerative Colitis

At different centers, eighty-nine patients with UC participated in a randomized placebo-controlled double-blind clinical trial. Patients (*n* = 44) consumed a dose of curcumin (1 g) two times per day together with sulfasalazine or mesalamine. On the other hand, other patients (*n* = 45) were given a placebo with sulfasalazine or mesalamine for six months. Out of the 89 patients, seven withdrew from the study. Thus, 82 patients were further subjected for evaluation. It revealed that the relapse rate was considerably greater in the placebo-treated group (20.5% (8/39)) when compared with the curcumin group (4.7% (2/43)). It was accompanied by a noticeable decrease in the suppression of clinical activity index (CAI) (*p* < 0.038) and endoscopic index (EI) scores (*p* < 0.0001) of the disease. After six months, the curcumin-treated group’s mean CAI improved from 1.3 to 1.0, whereas it raised from 1.0 to 2.2 in the placebo group. Furthermore, patients who consumed curcumin had considerably improved EI scores. This study was the first multicenter trial that showed promising results of using curcumin in patients with IBD [123].

Singla’s group conducted a double-blind pilot trial that included 45 patients with mild or moderately active UC and DAI scores of 3–9 with endoscopic disease extent up to 25 cm. They were administered NCB-02 (curcumin) enema and oral 5-aminosalicylates (5-ASA) or placebo enema and oral 5-ASA (control group). After eight weeks, the disease response was assessed by a decrease in UC and the DAI score, endoscopic activity progress, and disease remission. Treatment response was seen in 56.5% of patients in the NCB-02 group compared with 36.4% (*p* = 0.175) in the placebo group. In the NCB-02 group, the clinical remission was observed in 43.4% of the patients whereas and in 22.7% of the patients in the placebo group (*p* = 0.14). On the other hand, 52.2% of the patients exhibited improvement on endoscopy in the NCB-02 group compared to 36.4% of the patients in the placebo group (*p* = 0.29). Further, patients who had completed eight weeks of treatment were put to per-protocol analysis. It revealed that the NCB-02 group exhibited strong clinical response (92.9% vs. 50%, *p* = 0.01), remission (71.4% vs. 31.3%, *p* = 0.03), and better endoscopic results (85.7% vs. 50%, *p* = 0.04) [124].

A double-blind controlled clinical trial indicated that curcumin supplementation was fruitful for remission induction in UC patients with 5-ASA treatment. Overall, 50 patients with mild and moderate UC undergoing 5-ASA treatment were divided into two groups (supplementation with either curcumin (3 g/day) or placebo for four weeks). As a result, 14/26 (54%) curcumin group patients and 0/24 placebo group patients achieved clinical remission (Simple clinical colitis activity index (SCCAI) ≤ 2) at the fourth week (*p* = 0.01, OR, 42.2, 95% CI, 2.3 to 760). Clinical response: 65.3% in the curcumin group versus 12.5% in the placebo group. Endoscopic remission: 38% in the curcumin group versus 0% in the placebo group. These results suggest that curcumin upholds UC (mild-to-moderate) reduction clinically and endoscopically devoid of causing harmful effects [125].

Peterson et al. compared turmeric or curcumin extracts to placebo on 30 healthy subjects in a randomized placebo-controlled trial. Three turmeric tablets (*C. longa* (1000 mg) + piperine (1.25 mg)) or curcumin tablets (curcumin (1000 mg) + piperine (1.25 mg)) were advised to be taken orally with food two times per day (total 6000 mg daily). Microbiota analyses showed that the individual reactions to treatment were different. The patterns within the groups were almost the same for turmeric and curcumin. Though in the control group the total decrease in bacterial species was 15%, in the turmeric and curcumin groups, this amount increased by 7% and 69%. The study results confirmed that curcumin and turmeric changed the gut microbiota in the same way. However, curcumin can effect most of the detected variations seen in turmeric-treated subjects [126]. In another randomized double-blind clinical study, 70 UC patients with mild or moderate UC were advised to take 1500 mg of curcumin or placebo daily for eight weeks. The results showed that the CAI score was considerably higher in the curcumin group than in the placebo group. Moreover, curcumin treatment decreased the high-sensitivity C-reactive protein (hs-CRP) concentration and erythrocyte sedimentation rate (ESR) significantly compared to the control. Furthermore, it enhanced the life quality of UC patients [127].

The research performed by Banerjee et al. examined the effectiveness and safety of bioenhanced curcumin (BEC) as add-on therapy in effecting clinical and endoscopic reduction in UC patients (mild or moderate). Sixty-nine patients were categorized into two groups and treated with BEC (*n* = 34) and placebo (*n* = 35). After six weeks, 44.1% (15/34) and 35.3% (14/34) of the patients had clinical and endoscopic remission, correspondingly, compared to none in the placebo-treated group. Likewise, BEC-treated patients’ clinical response was considerably higher (18/34, 52.9%) than that of placebo-treated patients (5/35, 14.3%) (*p* = 0.001). At three months, the endoscopic remission, clinical response, and clinical remission rates were 55.9% (19/34), 58.8% (20/34), 44% (16/34) in the BEC group and 5.7% (2/35), 28.6% (10/35), 5.7% (2/35) in the placebo group. After six and 12 months, 95% (18/19) and 84% (16/19) of the BEC-treated patients maintained clinical remission. Collectively, the results showed that BEC is a safe option without adverse effects [128].

### 6.2. Crohn’s Disease

Holt et al. organized a small-scale pilot study to assess curcumin’s effectiveness in five CD patients. Patients received curcumin (550 mg) two times daily for the first month and then 550 mg of curcumin three times daily for the second month. Condition of all the five patients improved (*p* < 0.02), with decreases in associated medications in four patients. Similarly, in the first month, 360 mg of curcumin was advised to be taken two times per day. The dose of curcumin was increased in the second month, and the patients were advised to take 360 mg of curcumin three times per day. Among the five patients, four exhibited better results. The curcumin’s effect was confirmed by the dropped level of the CD activity index, with a mean decrease of 55 points, and the sedimentation rate with a mean of 10 mm/h decrease [129].

Researchers from Japan for the first time investigated the curcumin derivative theracurcumin’s effect in mild and moderate CD patients in a randomized double-blind clinical trial conducted at five different medical institutes in Japan. Theracurcumin (360 mg/day, *n* = 20) or placebo (*n* = 10) was advised to be taken for 12 weeks. The theracurcumin-treated group exhibited a considerable reduction in the clinical disease activity after 12 weeks. The clinical reduction observed at 4, 8, and 12 weeks was 35%, 40%, and 40%, respectively, which was considerably higher than in the placebo group. Theracurcumin also exhibited a decrease in the severity of CD and stronger endoscopic reduction levels in the theracurcumin group (15% compared to 0% in the placebo-treated groups), suggesting the potential of using theracurcumin to ameliorate the CD [130].

CD recurrence following surgery is one of the major worries. Thus, it is necessary to study the efficiency of curcumin in protecting against postoperative recurrence of CD. Recently, Bommelaer et al. performed a clinical trial on patients who underwent surgery for CD. This randomized controlled trial was conducted at eight referral centers in France with 62 individuals with CD undergoing bowel resection. They were advised to take 2.5 mg/kg of azathioprine and were arbitrarily allocated to either the oral curcumin group (3 g/day; *n* = 31) or the placebo (*n* = 31) group for six months. The results showed that the endoscopic recurrence (score ≥ i2) was seen in 18 patients (58%) of the curcumin-treated group and in 21 patients (68%) in the placebo group (*p* = 0.60). Forty-five percent of the patients featured clinical recurrence of CD in the placebo group as compared with 30% of the patients in the curcumin group (*p* = 0.80). This clinical trial was terminated after short-term examination because of futility. The study results stated that curcumin is not very effective in preventing CD recurrence post-surgery [131]. Table 5 shows the significant characteristics and the results from the clinical trials of curcumin in the treatment of IBD.

## 7. Opposing Voices against Curcumin

Rasyid et al. detailed that curcumin possibly leads to gallbladder reduction. Thus, patients who have gallstones or bile duct obstructions could not be recommended to use curcumin. The placebo-controlled study, including 12 healthy volunteers, shows that 20 mg curcumin supplementation results in a 29% decrease in the gallbladder size. It is significantly different from placebo [136]. A subsequent study by the same group showed that curcumin supplementation (40 and 80 mg) resulted in a 50 and 72% reduction in the gallbladder size, respectively [137]. Anemia often comes across in patients with IBD, compromising life and deteriorating the prognosis of the disease. The intestinal blood loss in inflamed mucosa and reduced dietary iron absorption is the primary factor for anemia’s pathogenesis in IBD. Curcumin is a potential candidate for IBD treatment, and many studies detailed the reason. We also discussed this in this section. However, curcumin has properties related to iron chelation. It can regulate the proteins involved in iron metabolism and reduce the spleen and liver’s iron content. Therefore, it can likely involve anemia progression and rigorousness of inflammation and iron deficiency in IBD. The curcumin effect was analyzed in the balance of iron in the DSS-induced colitis mice (C57Bl/6 and BALB/c) after supplying an iron-sufficient diet.

Curcumin supplementation produced mild anemia, aggravated colitis, reduced iron stores, and decreased the survival rate independent of the mouse strain. The study revealed that the use of curcumin has to be complemented through observing parameters of the erythroid cells to evade the increase in iron deficiency anemia in IBD [138]. Several questions have been raised about the curcumin^’^s protective effects in particular antioxidant activities. Curcumin uses prooxidative effects through increasing ROS levels [139]. Furthermore, preincubation with curcumin (5–50 μM) considerably increases doxorubicin-made ROS production and restrains glutathione reduction (sign of anemia) in cardiomyocytes at nontoxic doses. Curcumin shows antioxidative effects at minimum doses (100 or 200 mg/kg), whereas it encourages lipid peroxidation at higher doses (400 mg/kg) as shown via the increased amount of thiobarbituric acid reactive substances (TBARS) [140]. The study proved that minimum doses of curcumin enhance the endogenous antioxidant system, whereas higher doses of curcumin cause oxidative stress, proposing that a higher dose of curcumin could produce toxic adverse effects on the heart. Furthermore, in vivo and in vitro studies show that curcumin obstructs platelet aggregation. Ideally, it features an additive effect with antiplatelet drugs. Supplementation with curcumin and aspirin showed a 60% or 61.1% safety from thrombosis, individually [141,142,143]. Thus, the simultaneous usage of curcumin and anticoagulant or antiplatelet drugs should be handled carefully.

## 8. Conclusions and Future Outlook

IBD is a major disease worldwide; unremitting oxidative damage, inflammation, and apoptosis are considered the main causes for the progression of intestinal damage in IBD. Conventional drugs and biologics are shown as ideal for mucosal healing and upholding clinical remission. However, conventional drugs and biologics always cause harmful effects, a major drawback seen in IBD therapy. In this context, curcumin is a potential candidate for IBD treatment. In the past decades, a considerable amount of evidence (many in vitro, in vivo, and clinical studies) accumulated for curcumin’s effectiveness in IBD treatment. The existing studies paved the opportunities to conduct more research and discover superior drugs based on curcumin for IBD treatment. Many research investigations explored cytokine pathways (i.e., IL-6) to treat IBD. Curcumin has been reported to obstruct several cytokine pathways. Therefore, it is a primary natural compound with lower toxicity and a better safety profile. However, the limited well-organized human studies could confine its application in clinical trials. Notably, the major breakthrough for curcumin achieved via encapsulating curcumin into nanoformulations improved curcumin’s characteristics, such as solubility, bioavailability, stability, absorbance, and therapeutic potential. However, curcumin nanoformulations have not been tested for IBD in clinical studies. Some curcumin nanoformulations discussed in this review can challenge different signaling pathways associated with IBD. However, these experimental studies have been performed on preclinical animal models. Thus, a major drawback is our limited knowledge of curcumin nanoformulation-associated risks in humans. Therefore, new clinical studies are required to be performed to understand the potential benefits of curcumin nanoformulations and associated risks in IBD patients.

On the other hand, there are some opposing voices against curcumin. It exhibits adverse effects that are a bit serious, and the mechanism is unclear. Thus, studies are needed to explain its safety and value. So far, clinical studies concentrated only on the curcumin-treated patient’s clinical remission. Apart from that, researchers should focus on the bioavailability and detection of curcumin in the body. It helps to confirm how and how much curcumin is valuable to avert the relapse of IBD by transforming the patient’s diet in cutback days, controlling the mucosal inflammation in the acute phase. Eventually, the primary information found in clinical trials is increasing in amount and is very positive. Further prospective clinical studies of curcumin in the upcoming days could help us define its possible valuable part in IBD patients’ management procedure.

## Figures and Tables

**Table 1:** The major features of Crohn’s disease and ulcerative colitis.

S. No	Characteristics	Crohn’s Disease	Ulcerative Colitis	References
1	Lesion site	Infects any part of the gastrointestinal tract	Infection limited to the colon	Waugh et al. [33]; Vecchi Brumatti et al. [34]; Younis et al. [35].
2	Spreading type	Patchy	Continuous	
3	Mucosal inflammation	Entire wall and transmural	Limited to the epithelial mucosa	
4	Frequently involved site	Ileum	Rectum	
5	Bloody diarrhea	Less common	Very common	
6	Perianal complications	Common	Rare	
7	Cytokine inflammation	IFN-y, IL-12, and TNF-α	IL-1, IL-5, and IL-33	

**Table 2:** The major factors associated with the inflammatory bowel disease pathogenesis.

S. No	Major Factors	References
1	Excessive release of IL-6, IL-12, IL-23, and TNF-α	Badr-El-Din et al. [53]; Baumgart and Carding, [54]; Niess, [55]; Sanchez-Munoz et al. [56]; Abraham and Cho, [57]; Matricon [58]; Kim et al. [59]; Younis et al. [35]
2	Upregulation of Th1, Th2, and Th17	
3	Impaired pathogen recognition and epithelial integrity	
4	Defective macrophages	
5	Overactive dendritic cells	
6	Absence of Treg cells	

**Table 3:** Details of in vitro and animal studies of curcumin administration in inflammatory bowel disease treatment.

S. No	Author/Year	Study Title	Dose, Duration, and Route of Administration	Cell line/Animal Model	Main Findings
1	Salh et al., 2003 [82]	Curcumin attenuates DNB-induced murine colitis	Curcumin (0.25% concentration) supplied in the diet (five days before treatment and five days after induction)	C3H mice/DNBS colitis	Controlled the loss of body weight, reduced the severity of histological parameters and activity of MPO, IL-1β, NF-κB, and p38 MAPK
2	Jian et al., 2005 [98]	Preventive and therapeutic effects of NF-κβ inhibitor curcumin in rats colitis induced by trinitrobenzene sulfonic acid.	Curcumin supplied in the diet at 2.0% of concentration, 14 days	SPF Wistar rats/TNBS colitis	Curcumin improved the histological score and attenuated the NF-κB signaling, blockage of IĸB degradation decreased the IL-1 expression level and increased IL-10 level of expression. Effective against TNBS colitis in rats.
3	Jiang et al. 2006 [71]	Curcumin-attenuated trinitrobenzene sulphonic acid induces chronic colitis by inhibiting the expression of cyclooxygenase-2	Curcumin (30 and 60 mg/kg) intake every day, intraperitoneal injection, 14 days	Sprague–Dawley rats/TNBS colitis	Reduced the MPO activity and suppressed the COX-2, IFN-α, and TNF-α expression
4	Camacho-Barquero et al., 2007 [84]	Curcumin, a curcuma longa constituent acts on the MAPK p38 pathway modulating COX-2 and iNOS expression in chronic experimental colitis	Curcumin (50–100 mg/kg) intake every day, 14 days	Wistar rats/TNBS colitis	Curcumin led to the reduction in MAPK activity, which may result in the suppression of COX-2 and iNOS immune signals; no differences were observed in JNK. Effective against TNBS colitis in rats.
5	Larmonier et al., 2008 [67]	Protective effects of dietary curcumin in mouse model of chemically induced colitis are strain-dependent	Curcumin (2%) supplied in the diet for 14 days	NKT-deficient SJL/J mice (Th1-mediated inflammation) and BALB/c mice (mixed Th1/Th2 response)/TNBS colitis	The efficacy of curcumin in TNBS colitis varies in BALB/c and SJL/J mouse strains. The exact mechanism governing these differences is unclear, the results suggest that the therapeutic value of curcumin may differ depending on the nature of immune dysregulation in IBD.
6	Lubbad et al., 2009 [86]	Curcumin attenuates inflammation through inhibition of TLR-4 receptor in experimental colitis	Curcumin (100 mg/kg) supplied in the diet, five days	Sprague-Dawley rats/TNBS colitis	Curcumin suppressed the TLR-4 and NF-κB activity in the inflamed tissue. Effective against TNBS colitis in rats.
7	Epstein et al., 2010b [87]	Curcumin suppresses p38 mitogen-activated protein kinase activation, reduces IL-1b and matrix metalloproteinase-3, and enhances IL-10 in the the mucosa of children and adults with IBD	Curcumin (5, 10, 20 μM)	Colonic mucosal biopsy and colonic myofibroblast (CMF) cell cultures	Curcumin effectively suppressed the p38 MAPK activity and reduced the IL-1b and matrix metalloproteinase-3, but increased the IL-10 expression in the mucosa of children and adults with IBD
8	Midura-Kiela et al., 2012 [76]	Curcumin inhibits interferon-gamma signaling in colonic epithelial cells	Curcumin (98.05%; 0, 25, 50, and 75 µM) free of contaminating curcuminoids	T-84 cells and young adult mouse colonocyte cells	Curcumin obstructed the IFN-α signaling and induced the transcription of genes (i.e., CII-TA and MHC-II) and T cell chemokines (CXCL9, CXCL10, and CXCL11)
9	Motawi et al., 2012 [88]	Effects of curcumin and ginkgo biloba on matrix metalloproteinases gene expression and other biomarkers of inflammatory bowel disease	Curcumin was given at a concentration of 2.0% (wt/wt) (three days before the treatment and five days after induction)	Wistar rats/TNBS colitis	Reduced the MPO activity and pro-inflammatory cytokine TNF-α and attenuated the MMP-1, MMP-3, and TIMP-1 expression.
10	Miller et al., 2014 [89]	Curcumin: a double hit on malignant mesothelioma	Curcumin (0–50 µM) for 24–72 h	Mouse and human malignant mesothelioma (MM) cells and LP9/TERT-1	Curcumin effectively suppressed the inflammation-related genes (i.e., NF-κB, TLR, and IL-1β)
11	McFadden et al., 2015 [31]	The role of curcumin in modulating colonic microbiota during colitis and colon cancer prevention	98.05% of pure curcumin (8–162 mg/kg/daily, for 0.05% and 1% diets) free of contaminating curcuminoids	Il10−/− mice on 129/SvEv	Curcumin enhanced the survival rate and reduced the colon weight/length ratio while no changes were seen in the control. Besides, curcumin efficiently managed the age-linked reduction in alpha diversity and improved the histology of the colon, bacterial richness, and the lactobacilli level in AOM/Il102/2
12	Cooney et al., 2016 [45]	A combined omics approach to evaluate the effects of dietary curcumin on colon inflammation in the Mdr1a −/− mouse model of inflammatory bowel disease	0.2% of curcumin (≥ 94% curcuminoid content and ≥ 80% curcumin) added to the diet	Mdr1a −/− mouse model	The study showed the activation of α-catenin regulates the anti-inflammatory effects of curcumin in the Mdr1a−/− mouse colon. Curcumin reduced the immune response and improved xenobiotic metabolism and resolution of inflammation by the reduction in neutrophil migration and increase in barrier remodeling. Curcumin effectively downregulated the ERK, FN1, TNFSF12, and PI3K complex activated during inflammation.
13	Loganes et al., 2017 [92]	Curcumin anti-apoptotic action in a model of intestinal epithelial inflammatory damage	1 µM curcumin (0–24 h)	HT29 cells	Curcumin treatment of HT29 cells before the inflammatory stimulation of IFN-γ decreased the cell apoptosis rate. Curcumin-induced anti-apoptotic activity was associated with the decrease in the IL-7 secretion in the HT29 cells, but, surprisingly, no effect on the NF-κB signaling pathway was observed. Curcumin exhibited a minor effect on the phosphorylation of proteins in this inflammatory signaling pathway.
14	Yang et al., 2018 [93]	Comparative effects of curcumin and tetrahydrocurcumin on DSS-induced colitis and inflammatory signaling in mice	Oral administration of curcumin/tetrahydrocurcumin with 0.05% carboxymethyl (0.1 or 0.25 mmol/kg) for seven days	Male ICR mice/DSS colitis	Curcumin was effective against DSS-induced colitis in mice, suppressed the NF-κB and STAT3 activation, and reduced the COX-2 and iNOS expression
16	Yue et al., 2019 [95]	Curcumin ameliorates dextran sulfate sodium-induced colitis in mice via regulation of autophagy and intestinal immunity	Curcumin (5 mg/kg and 60 mg/kg) added to the diet, eight days	BALB/c mice/ DSS colitis	Curcumin administration effectively controlled the progress of the DSS-induced colitis in mice by regulating cytokine networks and obstructing excessive autophagy
17	Zhang et al., 2019 [96]	Curcumin and resveratrol suppress dextran sulfate sodium-induced colitis in mice	Curcumin (50 mg/kg) added to the diet, 14 days	BALB/c mice/DSS colitis	Curcumin and resveratrol effectively treat the experimental colitis in mice by attenuating the intestinal inflammatory cascade reaction, decreasing the autophagy, and controlling the signals of SIRT1/mTOR.
18	Wei et al., 2021 [97]	Curcumin ameliorates DSS‑induced colitis in mice by regulating the Treg/Th17 signaling pathway	Curcumin (100 mg/kg) added to the diet, seven days	Mice/DSS colitis	Curcumin showed the protective effects against DSS colitis in mice by mediating the re-equilibration of Treg/Th17 cells. The regulatory mechanism is possibly associated with IL‑23/Th17.

**Table 4:** Details of in-vitro and animal studies of curcumin combinations with other therapeutic molecules and modified curcumin formulations administration in inflammatory bowel disease treatment.

S. No	Author/Year	Study Title	Modified Curcumin Formulations/Curcumin with Other Therapeutic Molecules	Cell Line/Animal Model	Main Findings
1	Li et al., 2007 [99]	Liposomal curcumin with and without oxaliplatin: effects on cell growth, apoptosis, and angiogenesis in colorectal cancer	Liposomal curcumin with oxaliplatin	LoVo and Colo205 cells and nu/nu mice	Liposomal curcumin exhibited a dose-based inhibition in the growth of colon cell lines LoVo and Colo205. The in vivo studies revealed the efficiency of liposomal curcumin by attenuating the CD3 expression, vascular endothelial growth factor, and IL-8 in a mice model.
2	Yadav et al., 2009a [100]	Effect of cyclodextrin complexation of curcumin on its solubility and antiangiogenic and anti-inflammatory activity in rat colitis model	Curcumin-cyclodextrin complex	Sprague–Dawley rats, DSS colitis	Curcumin-cyclodextrin complex inhibited the activation of NF-κB and blockade of infiltration of inflammatory cells (CD4 and CD8 T cells). It attenuated DSS-induced colitis in rats.
3	Yadav et al., 2009b [101]	Novel formulation of solid lipid microparticles of curcumin for anti-angiogenic and anti-inflammatory activity for optimization of therapy of inflammatory bowel disease	Solid lipid microparticles of curcumin (curcumin SLM)	Sprague–Dawley rats, DSS colitis	The increase in body weight and colon length in curcumin SLM-treated rats when compared with native curcumin-treated and DSS control rats. It reduced the number of cells in the mucosa of the colon and effectively reduced the degree of colitis.
4	Gugulothu et al., 2014 [102]	pH-Sensitive nanoparticles of curcumin-celecoxib combination: evaluating drug synergy in ulcerative colitis model	Curcumin-celecoxib-loaded polymeric nanoparticles (CUR-CelNPs)	Sprague-Dawley rats TNBS colitis	CUR—CelNPs considerably reduced the MPO and LPO activity as well as increased the superoxide dismutase (SOD) activity when compared with curcumin or nanoparticles alone. The synergic effect of curcumin and celecoxib exhibits the better therapeutic effect in treating UC.
5	Beloqui et al., 2014 [29]	pH-sensitive nanoparticles for colonic delivery of curcumin in inflammatory bowel disease	Curcumin polymeric nanoparticles combining both poly (lactic-*co*-glycolic) acid (PLGA) and polymethacrylate	Caco-2 cells, C57BL/6 mice, DSS-induced colitis	Curcumin polymeric nanoparticles considerably decreased the secretion of TNF-α in LPS-stimulated macrophages. They effectively reduced the neutrophil infiltration and secretion of TNF-α and helped maintain the colon structure like in the control group in a DSS-induced colitis model.
6	Li et al., 2015 [105]	Curcumin—piperine mixtures in self-microemulsifying drug delivery system for ulcerative colitis therapy	Curcumin and piperine co-encapsulated into a nanoformulation (CUR-PIP-SMEDDS)	BALB/c mice (pathogen-free), DSS-induced colitis	More stable in colons, increased encapsulation. Use of CUR-PIP-SMEDDS showed a better anti-colitis activity in the inflamed colon region.
7	Mutalik et al., 2016 [106]	Development and performance evaluation of novel nanoparticles of a grafted copolymer loaded with curcumin	Curcumin polymeric nanoparticles	HCT116 cells Wistar rats, acetic acid-induced colitis	Curcumin polymeric nanoparticles showed a controlled and targeted release of curcumin as well as better absorption than when delivered as free curcumin. They effectively decreased the myeloperoxidase and nitrite levels. Furthermore, they prevented the loss of body weight and attenuated colonicinflammation.
8	Beloqui et al., 2016 [30]	A comparative study of curcumin-loaded lipid-based nanocarriers in the treatment of IBD	Curcumin—lipid based nanocarriers	J774 murine macrophages and Caco-2 cells, C57BL/6 mice, DSS-induced colitis	Increased curcumin retention at the intestinal site and permeability. Curcumin—lipid-based nanocarriers reduced the infiltration of neutrophils and secretion of TNF-α as well as exhibited efficiency for IBD treatment.
9	Huang et al., 2017 [109]	Effects of curcumin plus soy oligosaccharides on intestinal flora of rats with ulcerative colitis	Curcumin + soy oligosaccharides	Sprague–Dawley rats, DNCB-induced colitis	Combination of curcumin and soy oligosaccharide attenuated the TNF-α and IL- 8 activity and reduced the colonic mucosa inflammation and tissue damage
10	Ohno et al., 2017 [111]	Nanoparticle curcumin ameliorates experimental colitis via modulation of gut microbiota and induction of regulatory T cells	Curcumin nanoparticles (theracurmin)	HT29 cells, BALB/c mice, DSS-induced colitis	Curcumin nanoparticles decreased the disease activity index, considerably improved mucosal permeability and the histological colitis score. Furthermore, curcumin nanoparticles suppressed the NF-κB activation.
11	Qiao et al., 2017 [112]	Orally delivered polycurcumin responsive to bacterial reduction for targeted therapy of inflammatory bowel disease	Curcumin polymeric nanoparticles (polyethylene glycol) (PEG) and curcumin (CUR) linked by a disulfide bond)	Caco-2 cells, C57BL/6 mice, DSS-induced colitis	Better solubility and targeted drug delivery in the inflamed regions of the gut. Enhanced transmembrane permeability and bioavailability. Notably, curcumin nanoparticles reduced the progression of the colon disease and effectively protected mice from IBD.
12	Kesharwani et al., 2018 [113]	Site-directed non-covalent polymer-drug complexes for IBD: formulation development, characterization, and pharmacological evaluation	Curcumin polymeric nanoparticles (Ora-Curcumin-S)	HCT116 and HT29 cells, BALB/cJ mice, DSS-induced colitis	Ora-Curcumin-S exhibited better solubility and stability. It reduced the colitis-associated symptoms. Controlled the loss of body weight, improved the colon length, colon edema, and spleen weight in DSS-induced colitis.
13	Chen et al., 2018 [114]	Facile fabrication of bowl-shaped microparticles for oral curcumin delivery to ulcerative colitis tissue	Bowl-shaped microparticles loaded with curcumin	Mice, DSS-induced colitis	Prolonged drug release, better encapsulation efficiency, targeted delivery, and excellent hydrophilicity. Supplementation with BMPs loaded with curcumin alleviates UC well based on the DSS-induced mouse model.
14	Chen et al., 2019 [116]	Oral administration of colitis tissue-accumulating porous nanoparticles for ulcerative colitis therapy	Porous poly (lactic-*co*-glycolic acid) nanoparticles and pluronic F127 (PF127) loaded with curcumin	Raw 264.7 macrophages, mice, DSS-induced colitis	Better biocompatibility and cellular uptake rate of curcumin than those of porous curcumin-loaded nanoparticles without PF127 modification (porous nanoparticles). They also effectively obstructed secretion of important proinflammatory cytokines (i.e., IL-6, IL-12, and TNF-α) and ameliorated the symptoms of UC.
15	Sharma et al., 2019 [32]	Improved uptake and therapeutic intervention of curcumin via designing binary lipid nanoparticulate formulation for oral delivery in inflammatory bowel disorder	Curcumin-loaded solid binary lipid nanoparticles (C-SBLNs)	Guinea pigs, DSS-induced colitis	Control and stable release of curcumin, improved cellular uptake, and targeted delivery. Supplementation with C-SBLNs decreased the infiltration of leucocytes, oxidative stress, and secretion of TNF-α and helped to keep the structure of the colon healthy as compared to free curcumin.
16	Desai and Monim, 2020 [118]	Colon targeted bioadhesive pellets of curcumin and cyclosporine for improved management of inflammatory bowel disease	Polymeric nanoparticles (bioadhesive pellet cores of curcumin)	Wistar rats, acetic acid-induced colitis	Combining curcumin and cyclosporine exhibited synergistic effects of managing IBD. Controlled the loss of weight and enhanced the clinical response, macroscopic and microscopic parameters of induced colitis when compared to cyclosporine and native curcumin.
17	Oshi et al., 2020 [119]	Curcumin nanocrystal/pH-responsive polyelectrolyte multilayer core−shell nanoparticles for inflammation-targeted alleviation of ulcerative colitis	Curcumin nanocrystal polyelectrolyte (chitosan/sodium alginate/cellulose acetate phthalate as shells)	Mice, DSS-induced colitis	Targeted delivery of curcumin into the inflamed colon tissue and enhanced biodistribution in the stomach and small intestine. Improved effectiveness in reducing inflammation-associated indicators in a DSS-induced colitis mice model.
18	Luo et al., 2020 [120]	Genipin-cross-linked human serum albumin coating using a tannic acid layer for enhanced oral administration of curcumin in the treatment of UC	Tannic acid (TA)-coated, genipin (Gnp)-crosslinked human serum albumin (HSA)-encapsulated curcumin nanoparticles (TA/CUR-NPs)	Caco-2 cells, mice, DSS-induced colitis	Controlled curcumin release and increased the curcumin uptake in cells. Oral administration of TA/CUR-NPs obstructed the TLR4-linked NF-κB signaling pathway and reduced the colitis symptoms compared to the controls.
19	Huang et al., 2021 [121]	Oral nanotherapeutics with enhanced mucus penetration and ROS-responsive drug release capacities for delivery of curcumin to colitis tissues	Hydrogel (chitosan/alginate)-embedding pluronic F127—catalase/curcumin nanoparticles (P-CUR/CAT-NPs)	Raw 264.7 macrophages, FVB male mice, DSS-induced colitis	Improved the cellular uptake efficiency of macrophage cells, effectively reduced the symptoms, and suppressed secretion of the major proinflammatory cytokines
20	Rotrekl et al., 2021 [122]	Composites of yeast glucan particles and curcumin lead to improvement of dextran sulfate sodium-induced acute bowel inflammation in rats	Curcumin loaded into yeast glucan particles	Wistar rats, DSS-induced colitis	Curcumin loaded into yeast glucan particles decreased the activity of proinflammatory cytokines (i.e., TNF-α, IL-1β, and IL-6) and MMP activity as compared to native curcumin in DSS-induced colitis rats.

**Table 5:** Details of clinical trials of curcumin in the treatment of inflammatory bowel disease.

S. No	Author/Year	Study Title	Dose and Duration	Population and Study Design	Main Findings
1	Cheng, 2001 [132]	Phase I clinical trial of curcumin, a chemopreventive agent, in patients with high-the risk or pre-malignant lesions	500–8000 mg/day, three months	Twenty-five patients with the risk of premalignant lesions in Taiwan. Patients with resection of bladder cancer, oral leukoplakia, stomach metaplasia, cervical intraepithelial neoplasm, and Bowen’s disease enrolled in this clinical trial.	Curcumin could not be measured in lower doses. Serum concentration varied from 0.5 ± 0.11 mM and 1.77 ± 1.87 mM at 4000 mg/day. Curcumin could not produce any adverse effects when taken by oral administration for three months, up to 8000 mg/day.
2	Sharma et al., 2004 [133]	Phase I clinical trial of oral curcumin: biomarkers of systemic activity and compliance	0.45 to 3.6 g/day, four months	Fifteen patients with colorectal cancer resistant to chemotherapy participated in this clinical trial	Supplementation of curcumin was associated with mild diarrhea, and uptake of 3.6 g/day of curcumin results in a noticeable amount of the compound and conjugates in the urine and plasma. Curcumin supplementation obstructs the PGE2 production in blood leukocytes. Study results lead us to conclude that the systemic pharmacological properties of a daily dose of 3.6 g of curcumin are appropriate for its evaluation in the prevention of malignancies at sites other than the gastrointestinal tract and also help to optimize the clinical evaluation of curcumin in a Phase II chemoprevention or chemotherapy trial.
3	Holt et al., 2005 [129]	Curcumin therapy in inflammatory bowel disease: a pilot study	Curcumin (550 mg and 360 mg/day) two times for the first month and three times for the second month	Five CD patients; pilot study	CD activity index dropped, and the condition improved in the majority of the patients
4	Hanai et al., 2006 [123]	Curcumin maintenance therapy for ulcerative colitis: randomized, multicenter, double-blind, placebo-controlled trial	Curcumin (capsules) (2 g) + 1.5–3 g 5-ASA or 1–3 g sulfasalazine/day (*n* = 45) or placebo + 5-ASA/sulfasalazine (*n* = 44) (six months)	89 patients participated in this clinical trial. Multicenter, randomized, double-blind, placebo-controlled study	Seven patients did not complete the trial. After six months, the relapse rate was 2/43 (4.65%) in the curcumin group and 8/39 (20.51%) in the placebo group Curcumin improved the clinical activity index (*p* < 0.038). In the curcumin-treated group, it improved from 1.3 to 1.0. In contrast, it raised from 1.0 to 2.2 in the placebo group. Furthermore, patients who consumed curcumin had a considerably enhanced endoscopic index (*p* < 0.0001). Curcumin was well-tolerated and not associated with any side effects.
5	Singla et al., 2014 [124]	Induction with NCB-02 (curcumin) enema for mild-to-moderate distal ulcerative colitis—a randomized, placebo-controlled, pilot study	140 mg NCB-02 (curcumin extract) enema + oral 1.6 g 5-ASA/day (*n* = 28) or placebo enema + oral 1.6 g5-ASA/day (*n* = 22), eight weeks	Mild to moderate UC patients (*n* = 45). Pilot, double-blind, randomized, placebo-controlled study	Two and six patients from the NCB-02 group and the placebo group, respectively, did not finish the trial. Treatment response was seen in 56.5% of patients in the NCB-02 group compared with 36.4% (*p* = 0.175) in the placebo group. In the NCB-02 group, the clinical remission was observed in 43.4% of the patients whereas and in 22.7% of the patients in the placebo group (*p* = 0.14). After eight weeks, clinical response was 92.9% in the NCB-02 group versus 50% in the placebo group (*p* = 0.01). Clinical remission: 71.4% in the NCB-02 group versus 31.3% in the placebo group. Improvement of endoscopic activity: 85.7% in the NCB-02 group versus 50% in the placebo group (*p* = 0.04). No side effects.
6	Lang et al., 2015 [125]	Curcumin in combination with mesalamine induces remission in patients with mild-to-moderate ulcerative colitis in a randomized controlled trial	Curcumin (capsules) (3 g) + 4 g 5-ASA/day (*n* = 26) or placebo + 4 g 5-ASA/day (*n* = 24), one month	Mild or moderate UC patients (*n* = 50). Multicenter, randomized, double-blind, placebo-controlled study	Clinical remission: 54% in the curcumin group versus 0% in the placebo group. Clinical response: 65.3% in the curcumin group versus 12.5% in the placebo group. Endoscopic remission: 38% in the curcumin group versus 0% in the placebo group. No adverse effects.
7	Kedia et al., 2017 [134]	Low dose oral curcumin is not effective in induction of remission in mild to moderate ulcerative colitis: results from a randomized double a blind placebo-controlled trial	Curcumin (450 mg/day) + 2.4 g 5-ASA/day (*n* = 29) or placebo + 2.4 g 5-ASA/day (*n* = 33), eight weeks	Mild or moderate UC patients (*n* = 62). Single-center, double-blind, randomized, placebo-controlled study	The minimum dose of curcumin (450 mg/daily) was ineffective in UC patients. No significant changes were observed between the clinical remission and endoscopic remission rates of curcumin- and placebo-treated groups. Twenty-one patients withdrew from the study.
8	Peterson et al., 2018 [126]	Effects of turmeric and curcumin dietary supplementation on human gut microbiota: a double-blind, randomized, placebo-controlled pilot study	Turmeric tablets (*C. longa* (1000 mg) + piperine (1.25 mg)) and curcumin tablets (curcumin (1000 mg) and piperine (1.25 mg)); the subjects were advised to take three tablets orally with food two times per day (total 6000 mg daily)	UC patients (*n* = 30). Randomized, double-blind, placebo-controlled study	Microbiota analyses showed that the individual reactions to treatment were different. The patterns within the groups were almost the same in the turmeric group and the curcumin group. Though in the control group the total decrease in bacterial species was 15%, in the turmeric and curcumin groups, this amount increased by 7% and 69%. The study results confirmed that curcumin and turmeric changed the gut microbiota in the same way and suggested that curcumin can effect most of the detected variations seen in turmeric-treated patients.
9	Shapira et al., 2018 [135]	Of mice and men: a novel dietary supplement for the treatment of ulcerative colitis	Two Coltect (500 mg curcumin, 250 mg green tea, and 100 µg selenium) tablets two times daily for eight weeks	Mild or moderate UC patients (*n* = 20)	Combined treatment with curcumin, green tea polyphenol, and selenium showed better outcomes with reduced inflammatory symptoms and disease activity index in patients. Among the 20 patients, 14 patients (70%) were improved; nine patients (45%) featured complete remission and four patients (20%) showed marked improvement. One patient (5%) featured moderate improvement. The clinical active index showed considerable reduction at four and eight weeks (*p* < 0.001). There was no improvement in symptoms for two patients, and one patient was excluded after eight weeks. Besides, the uncertain condition resulted in exclusion of three subjects from the study. In eleven patients (69%), endoscopic improvement was observed, and four patients (25%) attained complete remission.
10	Sadeghi et al., 2020 [127]	The effect of curcumin supplementation on clinical outcomes and inflammatory markers in patients with ulcerative colitis	Curcumin (1.500 mg/day) + routine drugs (*n* = 35) or placebo + routine drugs (*n* = 35), eight weeks	Mild or moderate UC patients (*n* = 70). Double-blind, randomized, placebo-controlled study	Four patients from the curcumin group and three patients from the placebo group were excluded from the study. Clinical remission: 83.9% (curcumin) vs. 43.8% (placebo). Significant decrease in high-sensitivity C-reactive protein concentrations and erythrocyte sedimentation rate in the curcumin group as compared to the placebo group. The mean inflammatory bowel disease questionnaire (IBDQ)-9 score was increased in the curcumin group in comparison with the placebo group. Furthermore, curcumin improved life quality of UC patients.
11	Sugimoto et al., 2020 [130]	Highly bioavailable curcumin derivative ameliorates Crohn’s disease symptoms: a randomized, double-blind, multicenter study	Theracurcumin (360 mg/day, *n* = 20) or placebo (*n* = 10), 12 weeks	Mild or moderate CD patients (*n* = 30). Randomized, double-blind, placebo-controlled, multicenter study	After 12 weeks, the theracurcumin-treated group showed a significant reduction in clinical disease activity. The clinical reduction observed at 4, 8, and 12 weeks was 35%, 40%, and 40%, respectively, which was considerably higher than in the placebo group. Theracurcumin also exhibited a decrease in the severity of CD and stronger endoscopic reduction in the theracurcumin group (15% as compared to 0% in the placebo-treated groups), suggesting the potential of using theracurcumin to ameliorate CD.
12	Bommelaer et al., 2020 [131]	Oral curcumin no more effective than placebo in preventing recurrence of crohn’s disease after surgery in a randomized controlled trial	2.5 mg/kg azathioprine; subjects were arbitrarily allocated to the oral curcumin (3 g/day; *n* = 31) group and the placebo (*n* = 31) group for six months	CD patients (*n* = 60). Multicenter, randomized, double-blind, placebo-controlled study	Endoscopic recurrence (score ≥ i2) was seen in 18 patients (58%) of the curcumin-treated group whereas and in 21 patients (68%) in the placebo group (*p* = 0.60). Forty-five percent of the patients featured clinical recurrence of CD in the placebo group as compared with 30% of the patients in the curcumin group (*p* = 0.80). The clinical trial was terminated after short-term examination because of futility. The study results stated that curcumin is not very effective in preventing CD recurrence post-surgery.
13	Banerjee et al., 2020 [128]	Novel bioenhanced curcumin with mesalamine for induction of clinical and endoscopic remission in mild-to-moderate ulcerative colitis: a randomized, double-blind placebo-controlled pilot study	The standard dose of mesalamine was randomized to either 50 mg bio-enhanced curcumin (BEC) or an identical placebo twice daily	Mild or moderately active UC patients (*n* = 69). Randomized, double-blind, placebo-controlled, pilot study	After six weeks, 44.1% (15/34) and 35.3% (14/34) of patients had clinical and endoscopic remission, correspondingly, compared to none in the placebo-treated group. BEC-treated patients’ clinical response was considerably higher (18/34, 52.9%) than that of placebo-treated patients (5/35, 14.3%) (*p* = 0.001). At three months, the endoscopic remission, clinical response, and clinical remission rates were 55.9% (19/34), 58.8% (20/34), 44% (16/34) in the BEC group and 5.7% (2/35), 28.6% (10/35), 5.7% (2/35) in the placebo group, respectively. After six and 12 months, 95% (18/19) and 84% (16/19) of the BEC-treated patients maintained clinical remission. No adverse effects.

## Data Availability

No new data were created or analyzed in this study. Data sharing is not applicable to this article.
